# Defining tumor-associated vascular heterogeneity in pediatric high-grade and diffuse midline gliomas

**DOI:** 10.1186/s40478-021-01243-1

**Published:** 2021-08-23

**Authors:** Xin Wei, Michaël H. Meel, Marjolein Breur, Marianna Bugiani, Esther Hulleman, Timothy N. Phoenix

**Affiliations:** 1 Division of Pharmaceutical Sciences, James L. Winkle College of Pharmacy, University of Cincinnati, Cincinnati, OH USA; 2 Research in Patient Services, Cincinnati Children’s Hospital Medical Center, Cincinnati, OH USA; 3 Princess Máxima Center for Pediatric Oncology, Utrecht, the Netherlands; 4 Department of Pediatric Oncology/Hematology, Cancer Center Amsterdam, VU University Medical Center, Amsterdam, the Netherlands; 5 Amsterdam Leukodystrophy Center, Amsterdam UMC, Amsterdam, The Netherlands; 6 Department of Child Neurology, Emma Children’s Hospital, Amsterdam UMC, Vrije Universiteit Amsterdam and Amsterdam Neuroscience, Amsterdam, The Netherlands; 7 Department of Pathology, Amsterdam UMC, Vrije Universiteit Amsterdam and Amsterdam Neuroscience, de Boelelaan 1117, 1081HV Amsterdam, The Netherlands; 8 Amsterdam Leukodystrophy Center, Amsterdam UMC, Amsterdam, The Netherlands

**Keywords:** Pediatric high-grade glioma, Diffuse midline glioma, Blood brain barrier, Endothelial cells, Neurovascular unit, Diffuse intrinsic pontine glioma, H3K27M, Wnt signaling

## Abstract

The blood–brain barrier (BBB) plays important roles in brain tumor pathogenesis and treatment response, yet our understanding of its function and heterogeneity within or across brain tumor types remains poorly characterized. Here we analyze the neurovascular unit (NVU) of pediatric high-grade glioma (pHGG) and diffuse midline glioma (DMG) using patient derived xenografts and natively forming glioma mouse models. We show tumor-associated vascular differences between these glioma subtypes, and parallels between PDX and mouse model systems, with DMG models maintaining a more normal vascular architecture, BBB function and endothelial transcriptional program relative to pHGG models. Unlike prior work in angiogenic brain tumors, we find that expression of secreted Wnt antagonists do not alter the tumor-associated vascular phenotype in DMG tumor models. Together, these findings highlight vascular heterogeneity between pHGG and DMG and differences in their response to alterations in developmental BBB signals that may participate in driving these pathological differences.

**Supplementary Information:**

The online version contains supplementary material available at 10.1186/s40478-021-01243-1.

## Introduction

The blood–brain barrier (BBB) is a specialized vascular structure within the brain formed by the neurovascular unit (NVU) which consists of endothelial cells, pericytes, astrocytes and neurons [1]. While essential for normal brain function and homeostasis, the BBB poses a problem for treating CNS related diseases since the majority of drugs and small molecules display limited brain penetration [2]. BBB function was historically considered disrupted in brain tumors based on studies using aggressive adult glioma models that do not accurately reflect the diversity and pathological heterogeneity identified across adult and pediatric brain tumor entities [3, 4]. Our understanding of intra- and inter-tumoral BBB heterogeneity continues to improve with advancements in defining molecular subgroups of human brain tumors, and the development of accurate patient-derived xenograft (PDX) and genetically engineered mouse models (GEMMs) that faithfully recapitulate features of primary human brain tumors.

Pediatric high-grade gliomas (pHGGs) are among the most common childhood brain tumors and can be divided into multiple subgroups based different features including histology, location, mutation status and molecular profile [5–8]. One of the most lethal pHGG types are H3K27M mutant diffuse midline gliomas (DMGs), which encompass midline and brainstem gliomas that harbor H3K27M mutations [7, 9]. Treatment options remain limited for DMG patients, and no chemotherapy or targeted therapy has demonstrated significant survival benefits thus far [8, 10]. One proposed reason for the failure of systemically delivered therapies is that DMGs maintains a more intact BBB compared to other non-brainstem tumors, as clinicians have noted that DMGs frequently display little to no contrast enhancement on magnetic resonance imaging (MRI) [11, 12]. Yet a detailed characterization or direct comparison of tumor-associated vasculature in cortical pHGGs and DMGs has not been systematically performed.

Here, employing patient derived xenografts (PDX) and in utero electroporation (IUE) based mouse models of cortical pHGG and brainstem DMG, we perform a histological and molecular analysis of tumor-associated vasculature. We show that cortical pHGGs induce changes traditionally associated with glioblastoma vasculature [13], including abnormal vessel morphology and BBB disruption, while brainstem DMGs maintain a similar vascular content and architecture to that of the normal brainstem. Additional transcriptomics studies support our phenotypic analyses and suggest that DMG endothelial cells are maintained in a stable and mature state. While expression of Wnt-antagonists in other aggressive pediatric brain tumors, such as medulloblastoma [14, 15] and adult glioblastoma induces BBB disruption [16, 17], we find that their expression does not alter the BBB or tumor-associated vascular phenotype in DMG models. Our findings provide direct evidence of tumor-associated vascular differences between DMG and pHGG tumors and underscore the need to consider intra- and inter-tumor heterogeneity in vascular content and BBB function when developing therapeutic strategies that target tumor cells or their vasculature.

## Materials and methods

### Plasmid constructs

DNA plasmids were construct as we previously described [18]. PBCAG**-**H3.3G34R-Ires-eGFP was generated by InFusion (Takara) PCR site directed mutagenesis from an H3F3A template plasmid (Addgene #42,632) and insertion into EcoR1 linearized PBCAG-Ires-eGFP. PBCAG-Dkk1-Ires-eGFP was generated by PCR amplification of Dkk1 and insertion by InFusion ligation into EcoR1 linearized PBCAG-Ires-eGFP. PBCAG-Fzd8-CRD-IgG was created by PCR amplification of Fzd8-CRD-IgG (Addgene #16,689) and insertion by InFusion ligation into EcoR1 linearized PBCAG-Ires-eGFP. All plasmids were verified by Sanger sequencing (CCHMC DNA sequencing core), and plasmid stocks prepared by using NuceloBond Xtra Maxi EF endotoxin-free kits (Machery-Nagel).

### IUE mouse models

All IUE related mouse work was done according to institutional and IACUC review boards (University of Cincinnati). The IUE procedure to generate pediatric high-grade glioma and diffuse midline glioma mouse models was performed as previously described [18]. Briefly, IUE pHGG mouse models were created by lateral ventricle injection of PdgfraD842V + H3.3G34R + DNP53 + Pbase plasmids (all at 1 µg/µL). IUE DMG mouse model were created by 4^th^ ventricle injection of PdgfraD842V + H3.3K27M + DNP53 + Pbase plasmids. Control BS or CTX IUE conditions were created by 4^th^ or lateral ventricle injections respectively of H3.3WT + DNP53 + Pbase plasmids. Survival curves were generated in GraphPad Prism using Log-rank Mantle-Cox statistical tests.

### Patient derived xenograft pHGG and DMG models and primary tumor samples

Pediatric HGG and DIPG/DMG patient derived xenograft (PDX) samples are from Dr. Esther Hulleman’s group at VU University Medical Center, Amsterdam, The Netherlands. All PDX experiments were carried out at the VU University Medical Center in accordance with the Declaration of Helsinki and national and institutional guidelines. 5- to 6-week old NOD/SCID/Il2rg-/- mice (Jackson) were intracranially injected with 500,000 cells of each of the following primary cultures: VUMC-DIPG-F [19], VUMC-DIPG-G, VUMC-HGG-11 [20], VUMC-HGG-14 [21]. The stereotactic coordinates that were used to inject into the pons (VUMC-DIPG-F and -G) were 0.8 mm laterally, 1 mm caudally, and 4.5 mm ventrally from the lambda. The stereotactic coordinates that were used to inject into the striatum (VUMC-HGG-11 and -14) were 2 mm laterally, 1 mm cranially, and 3 mm ventrally from the bregma. Cells were injected in an injection volume of 5 μL at a flow rate of 1 μL/minute to minimize the neurological side effects of the procedure. Primary tumor-tissue was obtained through surgical resection (pHGG) or via a brain autopsy study (DIPG/DMG) in the Amsterdam UMC (Amsterdam, the Netherlands), in accordance with the declaration of Helsinki and approved by the institutional review board of Amsterdam UMC, location VUmc (METc VUmc, study number: VUMC2009/237) and the Scientific Committee of the Dutch Childhood Oncology Group (DCOG).

### Tissue collection, processing and immunostaining

Upon development of neurological symptoms, mice were deeply anesthetized before perfusion with cold DPBS followed by 1% PFA. Brains were rapidly dissected in cold DPBS and then fixed in 100% MeOH at 4 °C overnight. Samples were rehydrated at 4 °C in DPBS for 4 h before embedding in 3% low-melt agarose gel (IBI scientific, #B70051). 150 um thick free-floating sections were cut using a Leica vibratome. Floating sections were incubated in blocking solution (PBS + 0.5% Triton x-100 + 10% normal donkey serum) at RT for 30 min before incubating with primary antibodies at 4 °C overnight. The next day sections were washed in DPBS, transferred to blocking solution containing the appropriate secondary antibodies (1:500) and incubated at room temperature for 2 h. Finally, sections were incubated in Hoechst (1:1000 in PBS) for 10 min before final DPBS washing and mounting onto slides (Fisher, Superfrost), and coverslipped (Prolong Gold Antifade, ThermoFisher). Primary antibodies used in this study include: eGFP (Aves, #GFP1020), CD31 (BD Biosciences, #550,274), Glut1 (Millipore, #07–1401), Collagen IV (BioRad, #161,115), Desmin (Cell Signaling, #5332), human Vimentin (eBioscience, #11–9897-82), Claudin-5 (Thermofisher, #352,588), Plvap (BD Biosciences, #550,274), Ter119 (Invitrogen #14–5921-82) and Hoechst (ThermoFisher). Corresponding secondary antibodies used were all purchased from Jackson ImmunoResearch. Images were acquired on a confocal microscope (Nikon A1), and image analysis was performed in NIH Image J. Statistical analyses of these data were performed in GraphPad prism as described in the manuscript. P-values of ≤ 0.05 were considered statistically significant.

For histology, mouse brain tissue was fixed in 10% formalin overnight and transferred to 70% ethanol before paraffin embedding. 5 μm thick sections were prepared on a microtome (Lecia), and processed for hematoxylin–eosin (H&E) staining. For immunohistochemistry of primary patient samples 5 µm thick paraffin sections were prepared and stained using standardized techniques. Primary antibodies used include anti-CD31 (Dako, #M0823), anti-Cldn5 (Invitrogen, #34–1600) and anti-Glut1 (Millipore, #07–1401). Stains were developed with secondary HRP and DAB immunoreactivity secondary kits (Dako) and counterstained with Hematoxylin before mounting.

### TMR dextran BBB permeability assay

10-kDa Tetramethylrhodamine (TMR)-dextran (ThermoFisher # D1817) was dissolved in sterile DPBS at a concentration of 10 mg/ml. TMR dextran was administered as previously described elsewhere [22]. Following circulation of the dextran tracer brains were harvested in ice-cold DPBS and fixed in 4% PFA overnight. Fixed brains were washed in DPBS the next day, followed by incubation in 30% sucrose for 48 h at 4˚C before embedding in tissue freezing media. 50 μm thick free-floating sections were cut using a Leica cryostat.

### Magnetic cell sorting (MACS)

Endothelial cells (ECs) were purified using magnetic sorting using MACS (Miltenyl Biotec) according to the manufacturer’s protocol. All antibodies, buffers and equipment were purchased from Miltenyl Biotec. Briefly, tumor or normal tissues were micro dissected under a fluorescent stereoscope, and 3–4 tumors or control tissue samples were pooled together to minimize variability between individual tumors. Samples were then dissociated by incubation in a Collagenase IV cocktail for 45 min at 37 °C. Collagenase IV dissociation solution was made by mixing 32 mg collagenase IV (Worthington, #LS004209), 10 mg Deoxyribonuclease I (Worthington, #LS002007), 20 mg Soybean trypsin inhibitor (Worthington, #LS003587) with 10 mL DPBS. Following incubation and trituration, cell suspensions were passed through 40 μm mesh cell strainers. Myelin and cellular debris were removed using debris removal solution (Miltenyl Biotec #130–109-398), and red blood cells (RBCs) removed by incubation in ACK lysing buffer (Thermo Fisher, #A10492-01). Cell pellets were resuspended in 90 μL PEB buffer / 10 million cells. PEB buffer was prepared by diluting MACS BSA stock solution (#130–091-376) 1:20 with autoMACS rinsing solution (#130–091-222). Cells were blocked by adding 10 μl of FcR blocking reagent and incubated with 15 μL of CD45 microbeads (#130–052-301) at 4 °C for 15 min, then applied to MS column (#130–042-201) against a magnetic separator followed by two washes with PEB buffer. CD45-negative flow through cells were then collected and labeled with CD31 microbeads (#130–097-418) and passed through a new MS column against a magnetic separator followed by two washes with PEB buffer. CD45-negative/CD31-positive ECs retained within the MS column were eluted in PEB buffer by expelling with the provided column plunger. Purified cell numbers were determined using an automated cell counter (Thermo Fisher) and then processed for total RNA isolation.

### RNA preparation, real-time quantitative PCR, whole transcriptome sequencing and analysis

Total RNA was isolated from freshly isolated samples using the NucleoSpin Plus RNA kit (Macherey–Nagel) as previously described [18]. cDNA was synthesized using SuperScript Vilo cDNA synthesis kit (Thermo Fisher, #11,754,050) according to manufacture protocol. Real-time PCR was performed using a Bio-Rad CFX qPCR system. The fold increase was determined using the 2 − ΔΔCT method. *Gapdh* was used as endogenous control to normalize mRNA expression level. The following primers were purchased from IDT. *Gapdh*, Fwd: AGG TCG GTG TGA ACG GAT TTG, Rvs: TGT AGA CCA TGT AGT TGA GGT CA; *Cd31*, Fwd,: ACG CTG GTG CTC TAT GCA AG, Rvs: TCA GTT GCT GCC CAT TCA TCA; *Tie2*, Fwd: GAG TCA GCT TGC TCC TTT ATG G, Rvs: AGA CAC AAG AGG TAG GGA ATT GA; *Vegfr2*, Fwd: TTT GGC AAA TAC AAC CCT TCA GA, Rvs: GCA GAA GAT ACT GTC ACC ACC; *NeuN*, Fwd: ATC GTA GAG GGA CGG AAA ATT GA, Rvs: GTT CCC AGG CTT CTT ATT GGT C; *Cd68*, Fwd: TGT CTG ATC TTG CTA GGA CCG, Rvs: GAG AGT AAC GGC CTT TTT GTG A; *Dkk1*, Fwd: CTC ATC AAT TCC AAC GCG ATC A, Rvs: GCC CTC ATA GAG AAC TCC CG. RNA-sequencing was performed as previously described [18]. RNA quality control was performed on a bioanalyzer (BioRad) to ensure the quality of each sample submitted. For isolation of polyA RNA, a NEBNext Poly(A) mRNA Magnetic Isolation Module (New England BioLabs) was used for polyA RNA purification with a total of 1 μg good quality total RNA as input. The SMARTer Apollo NGS library prep system (Takara) was used for automated polyA RNA isolation. For RNA sequencing library preparation, the library for RNA-seq was prepared by using the NEBNext Ultra II Directional RNA Library Prep Kit (New England BioLabs). After indexing via PCR enrichment (8 cycles), the amplified libraries together with the negative control were cleaned up for quality control analysis. To study differential gene expression, individually indexed and compatible libraries were proportionally pooled (~ 25 million reads per sample in general) for clustering in the cBot system (Illumina). Libraries at the final concentration of 15 pM were clustered onto a single-read flow cell using the IlluminaTruSeq SR Cluster Kit v3, and sequenced to 51 bp using theTruSeq SBS Kit v3 on the Illumina HiSeq system. Sequence reads were aligned to the reference genome using the TopHat aligner and reads aligning to each known transcript were counted using Bioconductor packages for next-generation sequencing data analysis. The differential expression analysis between different sample types was performed using the negative binomial statistical model of read counts as implemented in the edgeR Bioconductor package. Transcriptional profiles were interrogated with iGEAK (Interactive Gene Expression Analysis Kit for microarray and RNA-seq data), an R (v3.3.2) and JavaScript based open-source desktop application [23]. Functional enrichment of differentially expressed gene lists between conditions was performed using iGEAK and gProfiler using default settings [24]. Transcription factor protein–protein interaction networks were generated using Enrichr [25]. All RNA-seq files are deposited in Gene Expression Omnibus as GSE179372.

## Results

### pHGG and DMG patient derived xenografts display tumor-associated vascular differences.

**Fig. 1:**
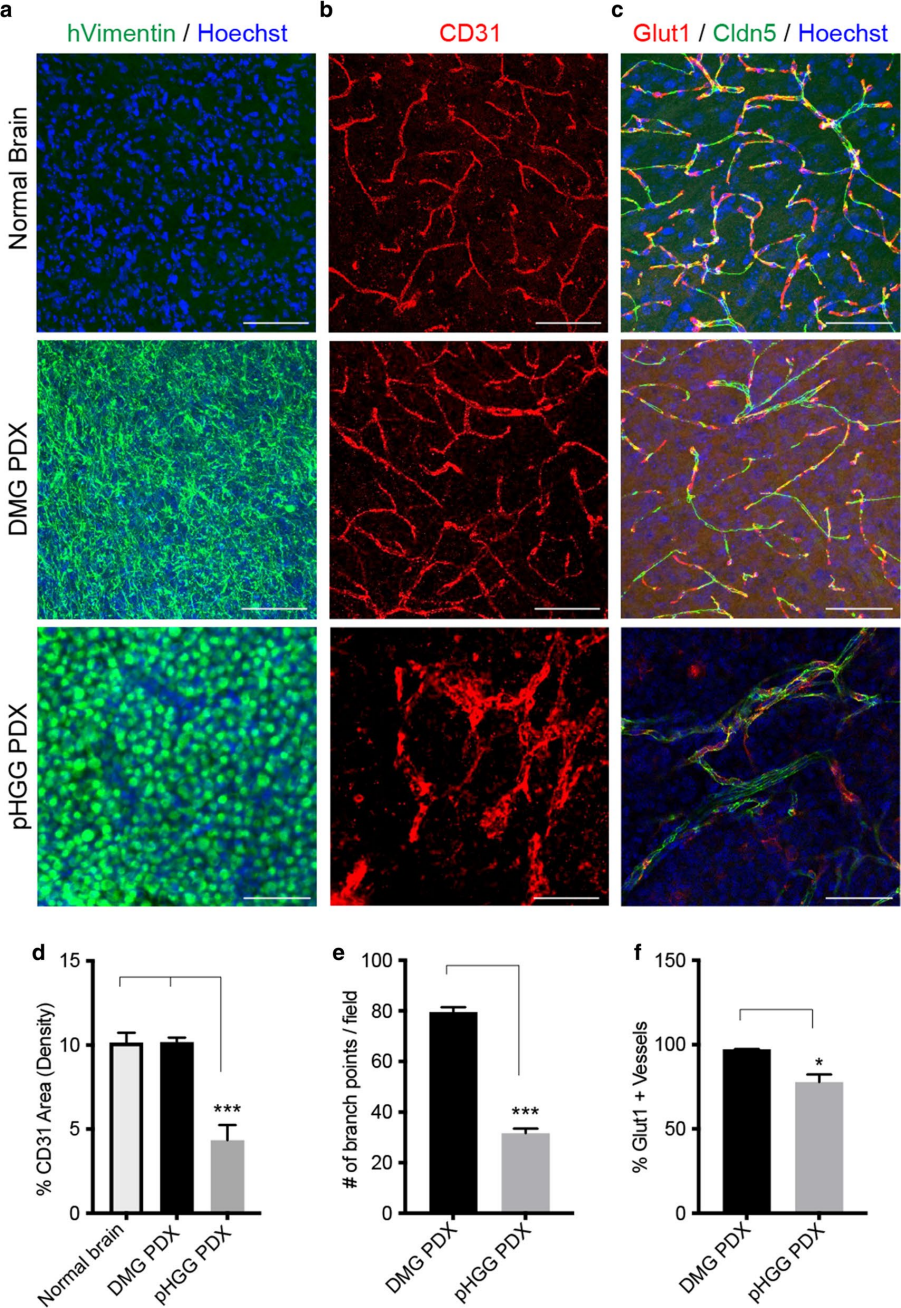
Tumor associated vascular differences in orthotopic patient derived xenograft pHGG and DMG models. Representative immunofluorescent images of pHGG, DMG PDX samples and normal brain labeled with **a** hVimentin and Hoechst, **b** CD31 and **c** Claudin-5 and Glut1 and Hoechst. Scale bar = 20 μm. Quantification of **d** CD31-positive blood vessel density in the normal brain (n = 5), DMG PDX (n = 5) and pHGG PDX (n = 3), **e** branchpoints and **f** % Glut1-positive blood vessels in DMG PDX (n = 5) and pHGG PDX (n = 3). Data are presented as mean ± SEM *P < 0.05, **p < 0.001, ***p < 0.0001, unpaired t-test with Mann–Whitney posthoc comparison

The presence of contrast enhancement (CE) in magnetic resonance imaging (MRI), indicating gadolinium leakage outside of blood vessels, is a common feature in most glioblastomas [13]. Yet prior radiological studies have noted DMG patients tend to display limited to no CE, suggesting the maintenance of a mostly intact BBB [11, 12, 26]. To investigate potential vascular differences between DMG and pHGG tumors, we examined available orthotopic PDX models [19–21]. Staining PDX tumor samples with a human specific tumor cell marker (hVimentin) and pan-endothelial marker (CD31) revealed minimal changes in the vasculature phenotype of DMG PDXs (Fig. 1a, b). In contrast pHGG PDXs displayed decreased blood vessel density, vascular branchpoints and enlarged lumens (Fig. 1b, d, e). Staining for the tight junction marker Claudin5 (Cldn5) and BBB associated glucose transporter Glut1 (also known as Slc2a1) found both expressed in pHGG and DMG PDX vasculature, although pHGG PDXs displayed small but significant decreases in Glut1-expressing vessels, along with regions of disorganized Cldn5 tight junctions in vessels (Fig. 1c, f). In contrast, DMG PDX blood vessels showed no noticeable differences in the expression or organization of Glut1 and Cldn5 compared to normal brain regions (Fig. 1c). In parts of pHGG tumors that contained necrotic regions there was a transition of Glut1 staining from vascular to non-vascular cells (i.e., tumor cells or associated macrophages), likely indicating a metabolic response to hypoxic conditions [27] (Additional file 1: Fig. S1). Additional staining of primary human DMG and pHGG samples for CD31, Cldn5 and Glut1 showed similar patterns to PDX models, providing further evidence for vascular differences between these tumors (Additional file 1: Fig. S2). This data, together with prior radiological studies in patients and PDX models [12, 19, 26, 28], suggests DMGs maintain a relatively intact BBB compared to cortical pHGGs.

### Cortical pHGG and brainstem DMG mouse models recapitulate tumor-associated vascular differences

**Fig. 2:**
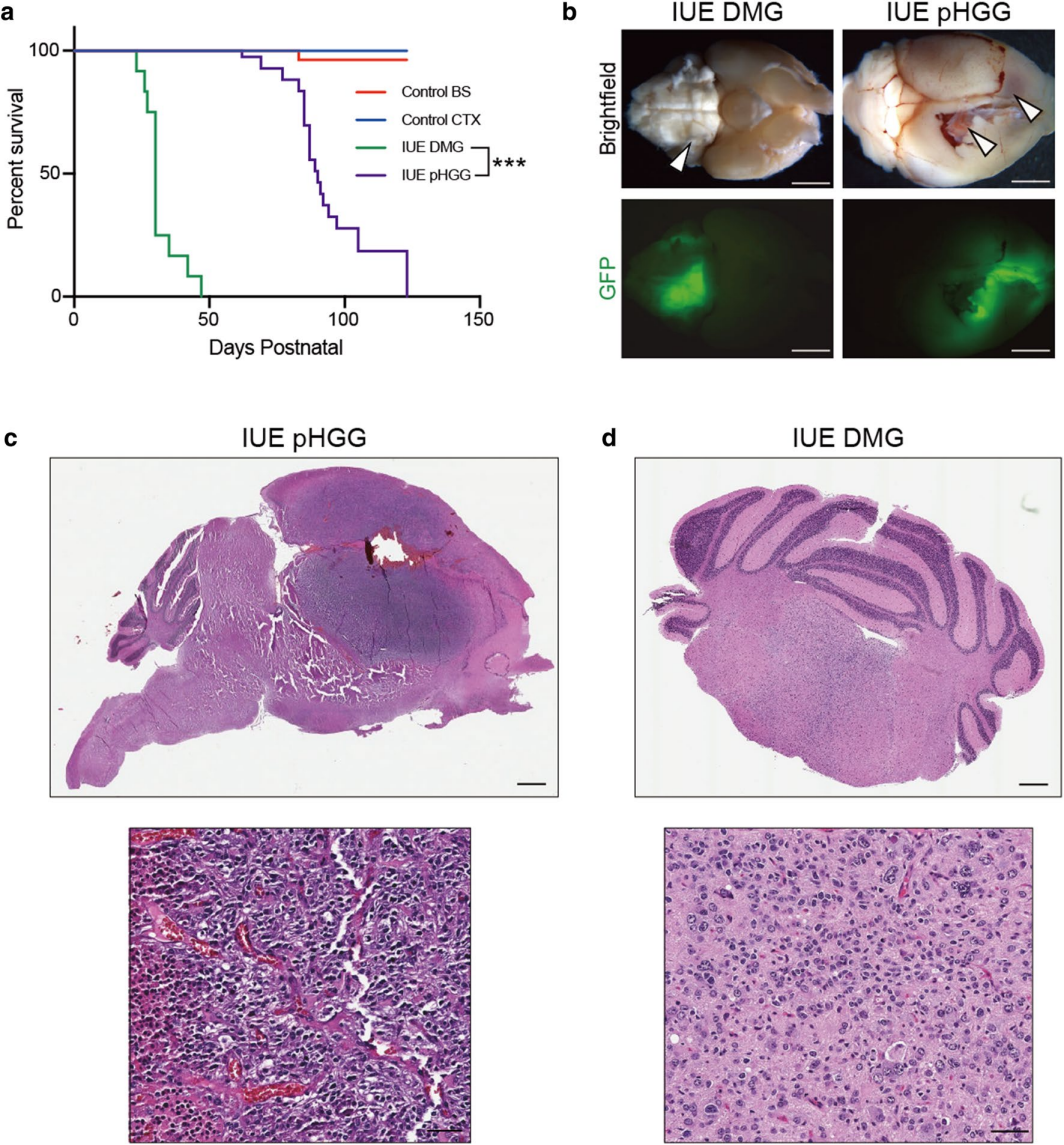
Generation of IUE pHGG and DMG mouse models. **a** Kaplan–Meier survival curves for IUE conditions: control brainstem (H3.3WT + DNp53, n = 11), control cortex (H3.3WT + DNp53, n = 12), IUE DMG (PdgfraD842V + DNp53 + H3.3K27M, n = 12) and IUE pHGG (PdgfraD842V + DNp53 + H3.3G34R, n = 19). ***p < 0.0001, Log-rank Mantel-Cox test. **b** Representative whole brain brightfield and GFP images depicting the regional location of IUE DMG and pHGG mouse models. Arrowheads point towards GFP-positive tumor regions. Scale bar = 1 mm. **c** H&E staining of IUE pHGG and IUE DMG sections. **d** High magnification inset images of H&E stained sections. Scale bars = 500 µm (top panels) and 50 µm (bottom panels)

To further examine vascular differences between pHGG and DMGs we employed recently developed glioma mouse models created by in utero electroporation (IUE) [18, 29]. DMG mouse models were made by brainstem targeted IUE of Piggybac DNA plasmids expressing PdgfraD842V, DNp53 and H3.3K27M. To generate cortical pHGG mouse models, we replaced H3.3K27M with a plasmid expressing H3.3G34R, combine with PdgfraD842V and DNp53 expressing Piggybac DNA plasmids. Both H3K27M DMG and H3G34R pHGG IUEs resulted in the generation of fully penetrant gliomas in successfully electroporated offspring. H3K27M IUE DMGs formed at a significantly shorter latency compared to H3G34R IUE pHGGs (median survival for IUE DMG model (n = 12) was 30 days and 79 days for HGG (n = 19) respectively; Log-rank Mantel-Cox test; *P* < 0.0001) (Fig. 2a). This is in agreement with prior studies demonstrating the ability of H3K27M mutations to accelerate glioma formation [18, 30, 31], and with recent data showing that the H3G34R mutation does not significantly alter the formation or latency of PdgfraD842V expressing gliomas [32]. Cortical pHGG mouse models displayed histological features of glioblastoma, including pseudopalisading necrosis and microvascular proliferation, while brainstem DMG models displayed features of grade III (3) high-grade gliomas (Fig. 2c, d). Control cortical and brainstem IUEs (DNp53 + H3.3WT) did not effectively drive gliomagenesis, with only one tumor arising out of all samples (n = 23) (Fig. 2a).

**Fig. 3:**
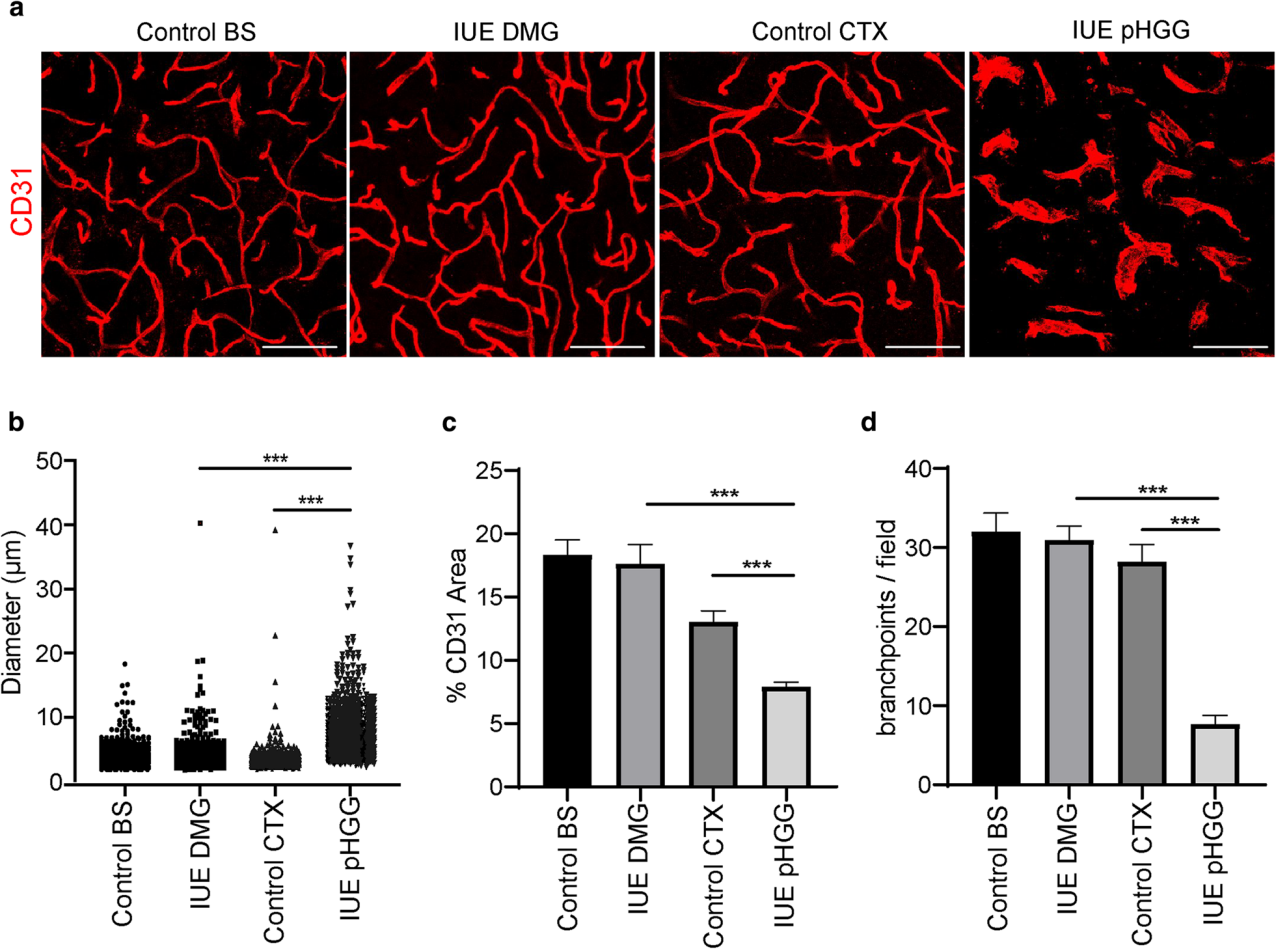
IUE pHGG and DMG mouse models recapitulate PDX tumor-associated vascular differences. **a** Representative immunofluorescent z-stack projection images of CD31-positive blood vessels in each experimental condition. Scale bar = 20 μm. Quantification of **b** CD31-positive blood vessel diameter, **c** density and **d** branch points. Control cortex and brainstem groups (n = 3), IUE DMG (n = 6) and IUE pHGG (n = 4). Data are presented as mean ± SEM. ***p < 0.0001; unpaired t-test with Mann–Whitney posthoc comparison

Upon collecting tumor samples, we noted areas of macroscopic hemorrhage in all the cortical pHGGs, a feature not found in brainstem DMG models (Fig. 2b). To compare tumor-associated vasculature within these models we stained normal control, IUE DMG and IUE pHGG tumors with the pan-endothelial marker CD31 and quantified blood vessel density, diameter and branchpoints. Relative to normal cortical vessels, IUE pHGGs displayed significantly enlarged and dilated vessels (Fig. 3a, b), reduced overall vascular density (Fig. 3a, c) and reduced vessel branching (Fig. 3a, d). While there was no significant difference between normal brainstem and IUE DMG blood vessels, comparison of IUE pHGG and DMG vessels revealed pHGG tumors displayed a mean vascular diameter approximately two times larger than that of DMG tumors, and reduced vessel density and branching of nearly two and five times smaller than that of DMG tumors respectively (Fig. 3a–d). IUE pHGG tumors do contain diverse intra-tumoral vascular features, with core regions showing tortuous and chaotic angiogenic vessels with diameters ranging from 2 μm to 40 μm, and bordering rim regions with a mixture of abnormal and normal vessel phenotypes. (Additional file 1: Fig. S3). Thus, de novo IUE mouse models of brainstem DMG and supratentorial pHGG recapitulate the architectural differences found in PDX tumor-associated vascular networks, providing an accurate system to study tumor-blood vessel interactions.

### pHGG and DMG mouse models display differences in vascular integrity and BBB function

**Fig. 4:**
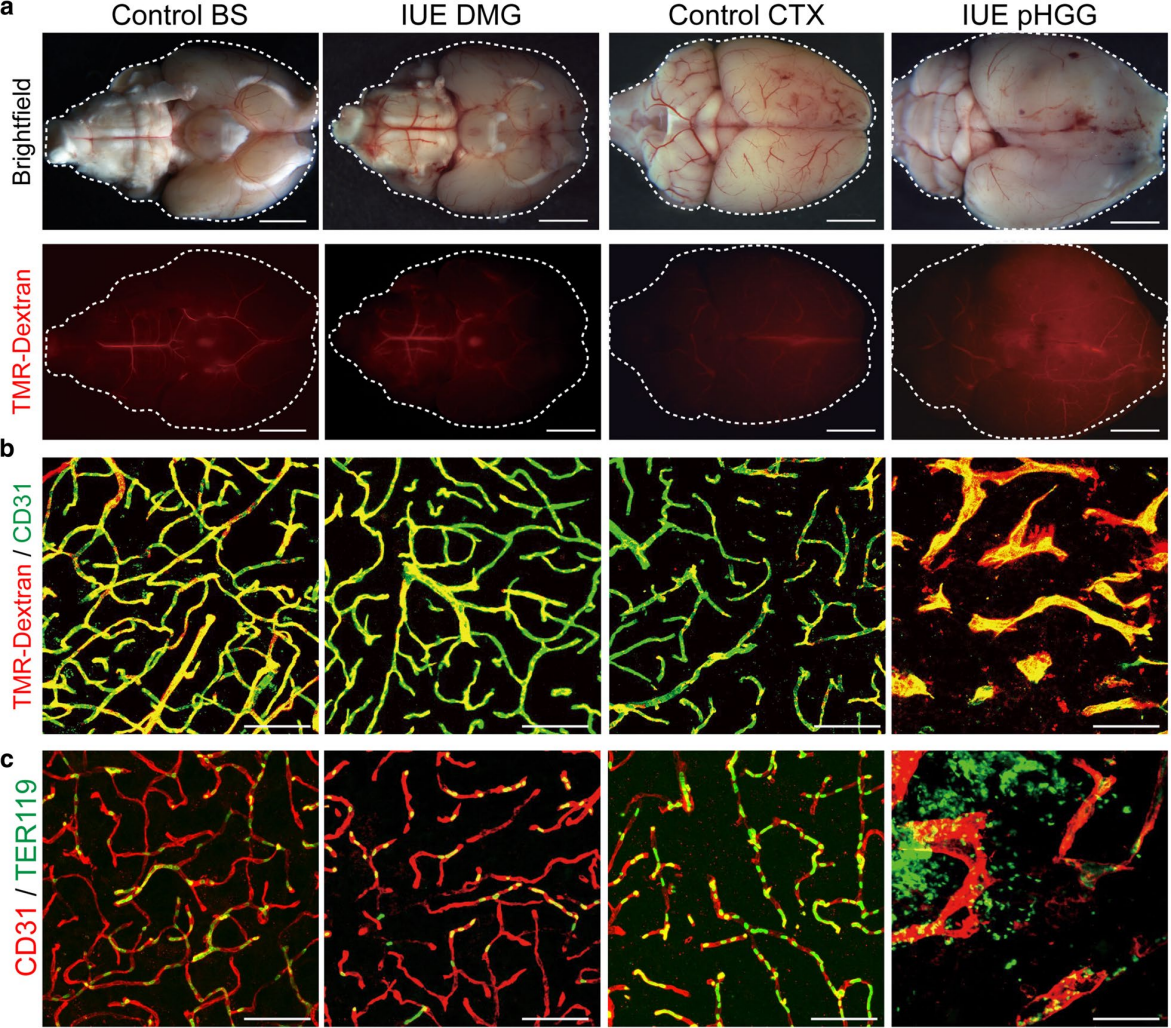
pHGG and DMG mouse models display differences in BBB function. **a** Representative whole brain images of brightfield and fluorescent TMR-dextran signal in control and IUE tumor conditions. Scale bar = 1 mm. **b** Representative immunofluorescent z-stack projection images of TMR-dextran and CD31 labeled sections. Scale bar = 20 μm. **c** Representative immunofluorescent z-stack projection images of control and IUE tumor conditions labeled with CD31 and Ter119 depicting the retention or extravascular leakage of red blood cells within samples. Scale bar = 20 μm

To gain additional insight into these vascular differences we examined the vascular permeability of control and IUE tumor models by circulation of a fluorescently labeled dextran tracer (10 kDa TMR-Dextran). No extravascular leakage was found in control brainstem and cortical samples, or in IUE DMG tumors (Fig. 4a, b). On the other hand, IUE pHGG tumors displayed extravascular dextran leakage, suggesting an increased level of vascular permeability (Fig. 4a, b). This was also supported by the presence of extravascular red blood cells in IUE pHGG, but not IUE DMG tumors, as visualized by TER119 staining (Fig. 4c).

**Fig. 5:**
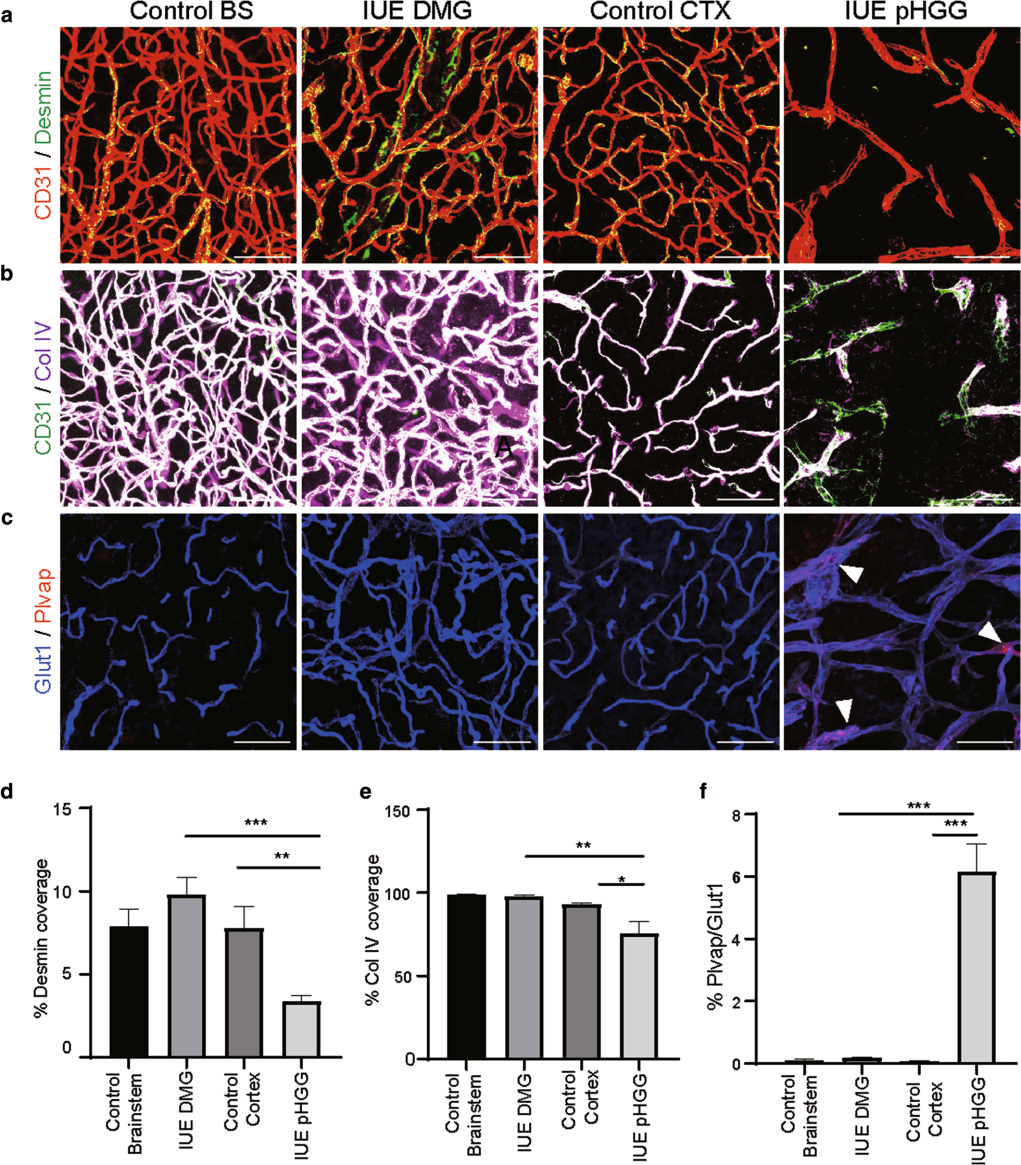
Mural cell coverage and BBB associated marker expression differences between pHGG and DMG mouse models. Representative immunofluorescent z-stack projection images of **a** Desmin and CD31, **b** Collagen IV and CD31, and **c** Plvap and Glut1 in each experimental condition. Scale bar = 20 μm. **d**, **e** Quantification of CD31-positive vessel coverage by desmin and collagen IV respectively. **f** Quantification of Plvap-positive area in Glut1-positive blood vessels in each condition. Data are presented as mean ± SEM. n = 3 for all conditions. *p < 0.05, **p < 0.001, ***p < 0.0001; unpaired t-test with Mann Whitney posthoc comparison

Interactions between endothelial cells and neighboring cell types which make up the neurovascular unit, (pericytes, astrocytes and neurons), are essential for instructing and maintaining blood–brain barrier function and vascular integrity. Immunostaining control and IUE glioma mouse models with Desmin, a marker of pericyte and smooth muscle cells, revealed no change in the vascular pericyte coverage in IUE DMG tumors, which maintained the same extent of pericyte coverage as the normal brainstem (Fig. 5a, d). IUE pHGG tumors displayed a significant decrease in pericyte investment compared to normal cortex and IUE DMGs (Fig. 5a, d). Changes in the extracellular matrix protein Collagen IV (ColIV) further demonstrated differences between IUE pHGG and DMG tumors, with IUE pHGG tumors showing decreased ColIV basement membrane staining, indicating changes in the neurovascular unit compared to normal brain and IUE DMG tumors (Fig. 5b, e). We also performed co-immunolabeling for Glut1 and Plasmalemma Vesicle Associated Protein (Plvap), a protein involved in endothelial fenestrae diaphragms, caveolae and trans-endothelial channels [33–35]. Plvap expression was not detected in Glut1-positive blood vessels in control brain regions or IUE DMG tumors, but could be found in some IUE pHGG vessels (Fig. 5c, f). While expression of Plvap could be sporadically found in pHGG blood vessels, its expression was not accompanied by the loss of Glut1, as previously described in medulloblastomas [14]. This would suggest at least the partial maintenance of a signaling program that regulates Glut1 expression in endothelial cells, rendering a hybrid or fluctuating state of BBB functionality. These data show that DMG blood vessels maintain numerous attributes of the normal NVU, while many of these elements are altered or partially disrupted in cortical pHGGs.

### Endothelial transcriptomes highlight differences and similarities between pHGG, DMG and normal brain endothelial signaling programs

**Fig. 6:**
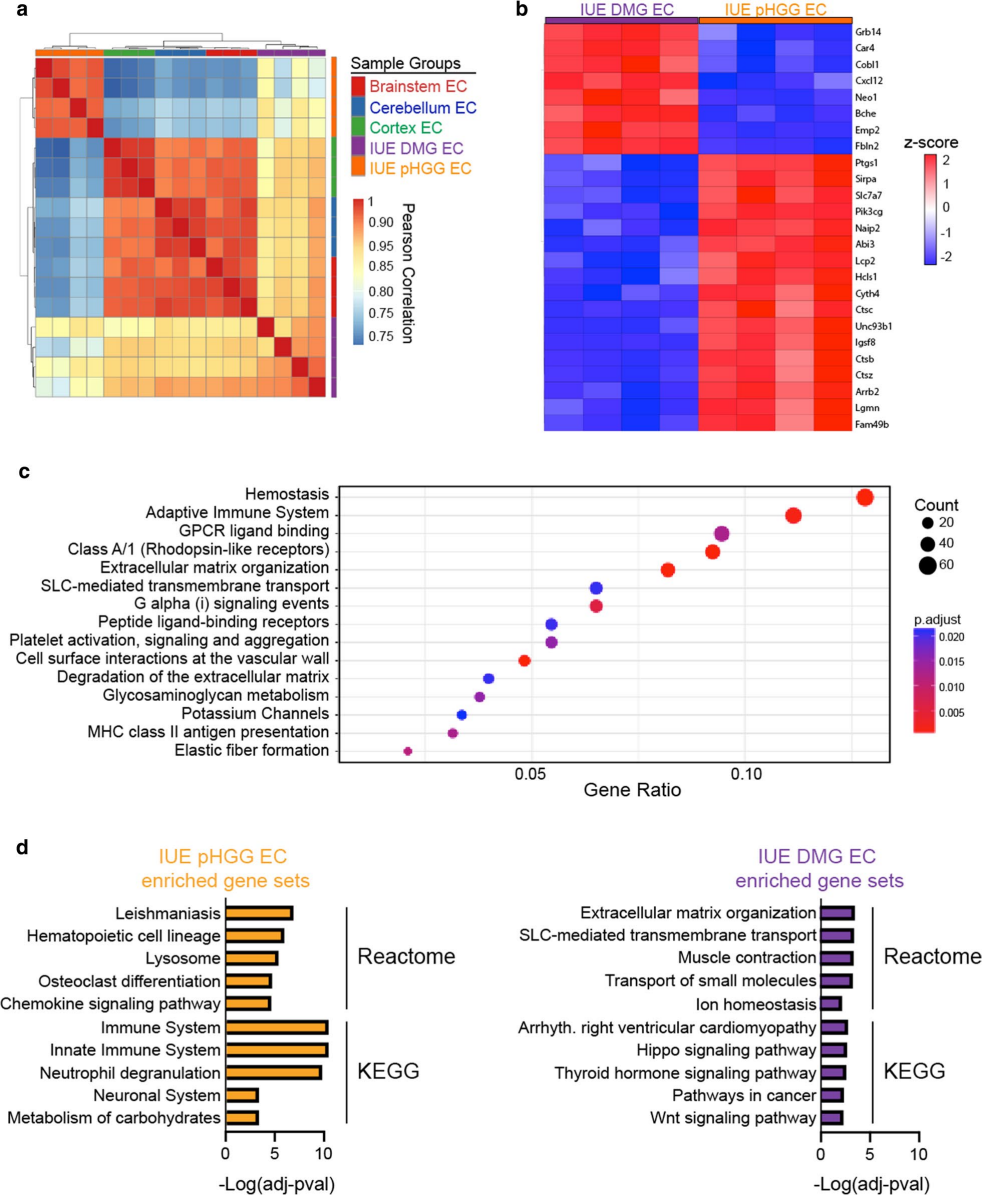
pHGG and DMG endothelial transcriptomes highlight heterogeneity of tumor-associated and normal brain endothelial signaling programs. **a** Hierarchical clustering of Pearson’s correlation plot visualizing the correlation values between samples. Scale bar represents the range of the correlation coefficients displayed. **b** Heatmap of the top 25 most significant (adj. p-value) differentially expressed genes between IUE DMG EC and IUE pHGG EC. **c** Gene sets enriched by over-representation analysis of differentially expressed genes (FC > 4, adj. *p* < 0.05) between IUE pHGG EC and IUE DMG ECs. **d** Gene sets enriched by over-representation analysis of differentially expressed genes (FC > 2, adj. *p* < 0.05) up-regulated in IUE pHGG EC or IUE DMG ECs

We next purified endothelial cells and vessel fragments from normal brain regions (cortex, cerebellum and brainstem) and IUE DMG and pHGG tumors to examine molecular differences by whole-transcriptome analysis. Using antibody labeled magnetic beads to isolate Cd31 + / Cd45- endothelial cells and vascular fragments, comparison of positive and negatively sorted populations from normal brain samples showed positive enrichment for endothelial (*Cd31*, *Tie2*, *Vegfr2*) and pericyte (*Pdgfrb*) genes, and negative enrichment for microglia (*Cd68*) and neuronal (*NeuN*, *Tub3*) genes (Additional file 1: Fig. S4). Following RNA-seq, hierarchical clustering by Pearson’s correlation of all samples revealed two main branches separating IUE pHGG EC from normal brain region ECs (BS, CB and CTX) and IUE DMG ECs. Further separation between normal brain ECs and IUE DMG EC groups was evident, with a second branch point dividing these groups (Fig. 6a). A similar pattern emerged by principal component analysis (PCA), with samples from each group clustering in the same general region, and IUE pHGG ECs segregating the furthest away from normal brain ECs (Additional file 1: Fig. S5). Comparison of differentially expressed genes (FC > 4, adj. p-val < 0.05) between IUE DMG EC and IUE pHGG EC identified over-represented gene sets related to immune system interactions (adaptive immune system, MHC class II antigen presentation), extracellular matrix regulation (ECM organization, ECM degradation), and vascular interactions (cell surface interactions at the vascular wall, platelet activation signaling and aggregation) (Fig. 6b, c). Further analysis of differentially up-regulated genes (FC > 2, adj. p-val < 0.05) in IUE pHGG ECs displayed enrichment in pathways related to immune response related pathways, while those up-regulated in IUE DMG ECs included extracellular matrix organization, SLC-mediated transmembrane transport, and signaling pathways (Hippo, Wnt) associated with BBB function [36–39] (Fig. 6d, Additional file 2: Table 1). Thus, beside preserving their morphology and blood–brain barrier function, IUE DMG blood vessels appear to maintain transcriptional programs that closely align with normal brain endothelium.

### Expression of secreted Wnt-antagonists does not alter DMG vascular phenotype

**Fig. 7:**
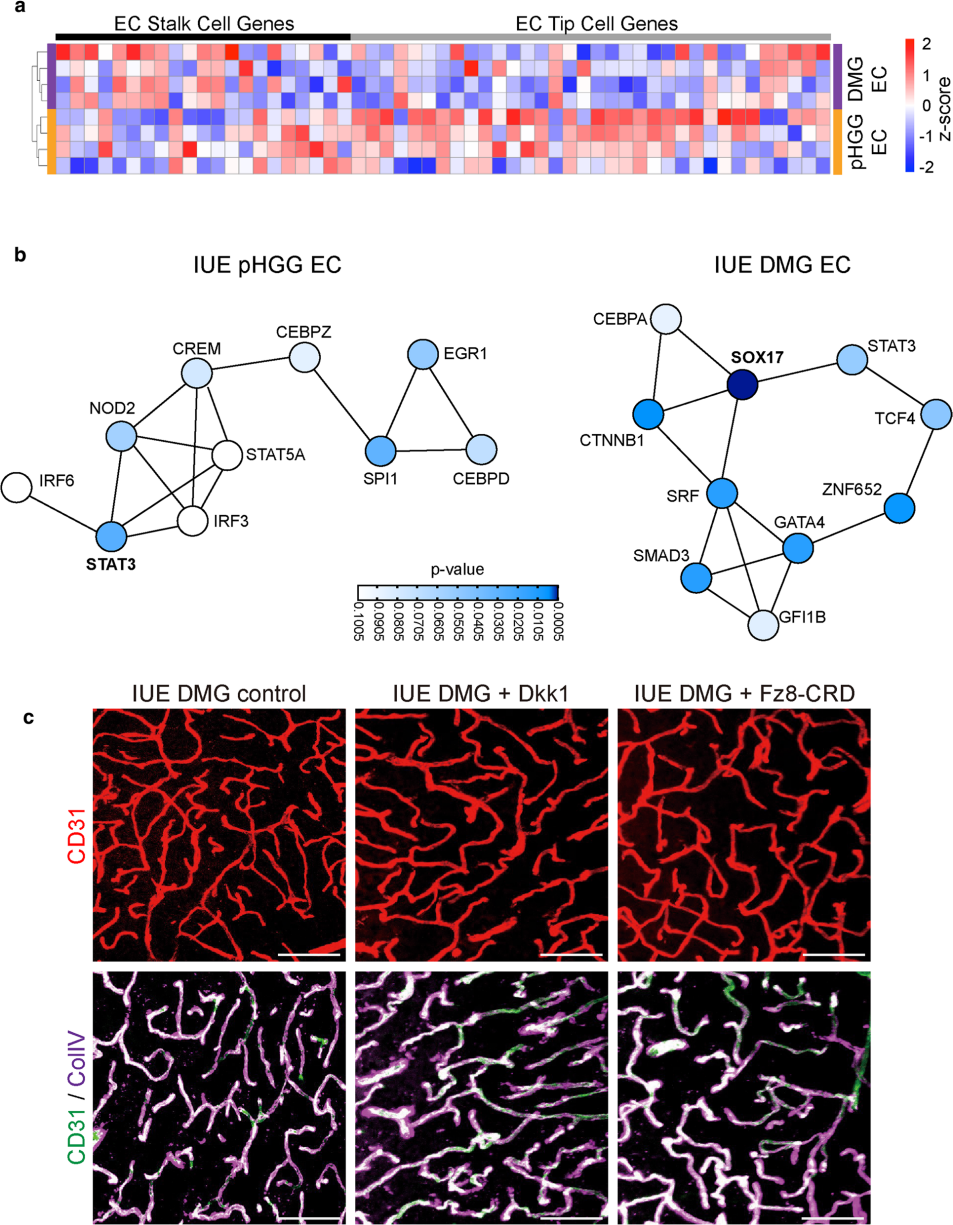
Expression of secreted Wnt-antagonists does not alter DMG vascular phenotype. **a** Heatmap of endothelial stalk and tip cell associated gene expression in IUE pHGG and DMG ECs. **b** Transcription factor protein–protein interaction networks enriched in IUE pHGG or DMG ECs. **c** Representative immunofluorescent z-stack projection images of CD31 labeled or CD31 and Collagen IV labeled blood vessels in IUE DMG control, Dkk1, or Fzd8-CRD-IgG tumors. Scale bar = 20 μm

DMGs are invasive brain tumors, and our vascular analyses indicate minimal disruption to established blood vessels within brain regions harboring tumor cells. This is corroborated by the expression pattern of genes associated with endothelial tip or stalk cell identity [40, 41]. IUE DMG ECs display increased expression of stalk cell genes, while IUE pHGG ECs show higher expression of tip cell genes (Fig. 7a). Further supporting the idea of a more mature and stable vascular state in DMGs, analysis of transcription factor protein–protein interaction (PPI) networks identified *Sox17*, a transcription factor (TF) that is highly expressed in mature brain endothelial cells [42], as the most significantly enriched TF in DMG ECs (Fig. 7b). *Sox17* is a positive inducer of Wnt signaling [42], and together with *Ctnnb1*, which was also enriched in DMG ECs (Fig. 7b), may promote stability through the maintenance of proper Wnt signaling levels. Within IUE pHGG ECs many enriched TFs in the PPI were associated with immune responses. These included Stat3, which was the most significantly enriched TF, and interferon response factors (IRF3/6) and NOD2, all of which can drive downstream signaling related to immune system activity.

Canonical Wnt-signaling is essential for blood–brain barrier formation in the developing brain [36, 37]. In addition, prior work in medulloblastoma and adult glioblastoma have shown that inhibition of endothelial Wnt-signaling, by either expression of secreted Wnt-antagonists such as Dkk1 and Wif1 [14, 16], or genetic deletion of Wnt signaling components in endothelial cells [17], results in tumor vascular abnormalities and blood–brain barrier dysfunction. To test whether Wnt antagonists could alter the vascular phenotype of DMG tumor models we expressed the Wnt receptor antagonist Dkk1, or a secreted version of the Fzd8 receptor (Fzd8-CRD-IgG) [43] in our IUE DMG mouse model (Additional file1: Fig. S6). IUE DMG tumors expressing empty vector control or the secreted Wnt antagonist (Dkk1 or Fzd8-CRD-IgG) developed tumors with similar latencies, and analysis of vascular content and supporting components, such as ECM proteins, did not identify any significant changes induced by secreted Wnt antagonist (Fig. 7c). Our data suggests that differences in the angiogenic state of tumor-associated vasculature will influence how they respond to other external cues, adding an additional layer of complexity to interactions within the tumor microenvironment.

## Discussion

**Fig. 8:**
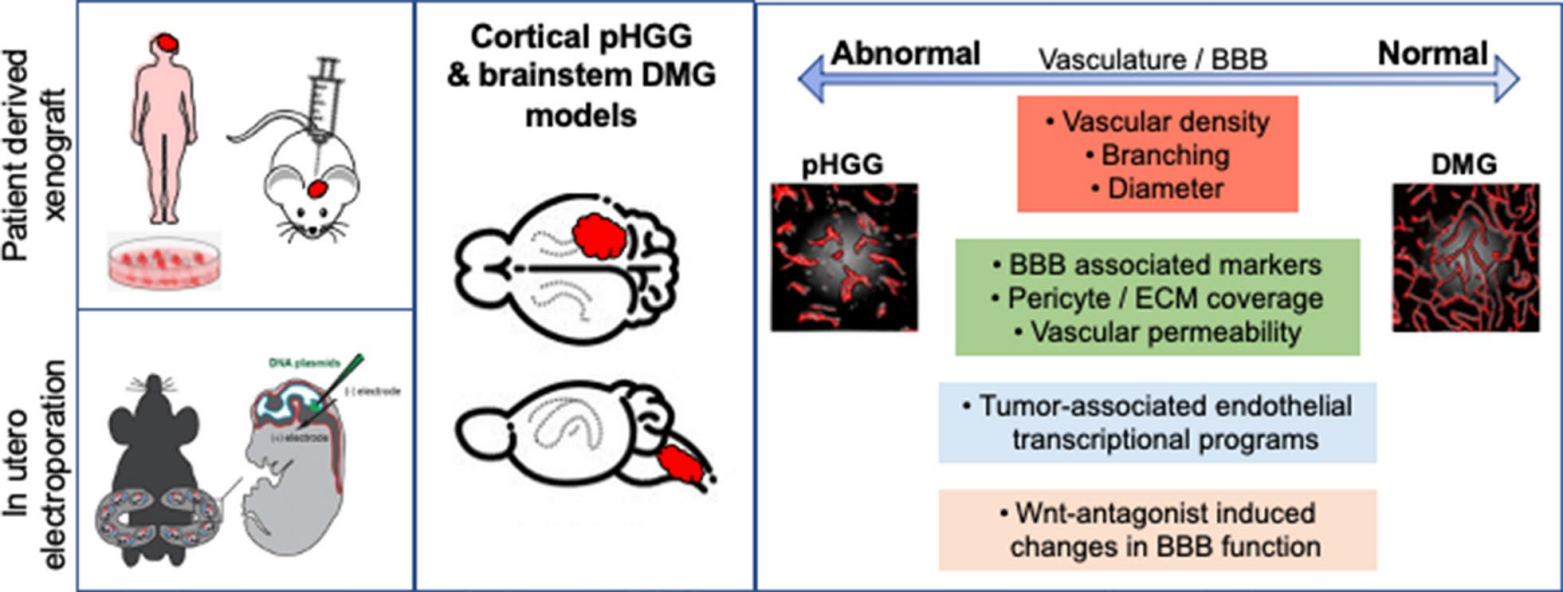
Summary of pHGG and DMG models and vascular phenotypes associated with each tumor type. Patient derived xenografts and in utero electroporation based cortical pHGG and brainstem DMG models were utilized to investigate in vivo vascular phenotypes. Across model systems, DMG tumor-associated blood vessels consistently displayed vascular phenotypes, BBB function and transcriptional programs similar to normal brain endothelium. pHGG tumor-associated blood vessels were associated with abnormal vascular phenotypes, BBB dysfunction, and transcriptional changes

Our analyses across pHGG and DMG implant based PDX and native forming IUE mouse models reveal phenotypic and molecular differences in tumor-associated vasculature, which recapitulate findings in biopsy and autopsy derived patient specimens. While variations in glioma BBB function have been appreciated within the field, including regional differences in glioma mouse models [44], a detailed comparison that catalogs and validates these differences between malignant gliomas has not been carried out. We show that the vascular network within DMGs remains mostly intact with respect to blood vessel morphology, BBB function and transcriptional programs, while cortical pHGGs display both phenotypic and transcriptional changes related to disorganized angiogenesis, inflammation and BBB dysfunction (Fig. 8). Moreover, DMG tumors display limited sensitivity to the expression of secreted Wnt antagonists, which have been shown to drive BBB dysfunction in glioblastoma and medulloblastoma [14, 16], suggesting heterogeneity in the response of tumor-associated blood vessels to extrinsic signals in the tumor microenvironment.

In pathological conditions, including brain tumors, the BBB presents a conundrum for treatment strategies. On one hand, the BBB is commonly cited as an important factor in brain tumor treatment resistance since the majority of drugs and small molecules display limited BBB penetration [3]. On the other hand, poor perfusion in abnormal and “dead end” vascular structures that lack BBB function can impede drug delivery [45]. Studies examining angiogenesis and BBB specification during normal CNS development have identified endothelial Wnt signaling as an essential regulator [36, 37]. The vascular phenotype of Wnt mutants shares many common features with that in glioblastoma, including chaotic architecture, hemorrhaging, the formation of glomeruli structures and a lack of BBB functionality [36, 37, 46–49], suggesting a direct link between Wnt signaling disruption and brain tumor vascular abnormalities. Indeed, compared to normal brain and DMG ECs, pHGG ECs show a modestly decreased Wnt-signaling at the transcriptome level, indicating that alterations in this essential BBB signaling pathway likely participate in generating pHGG vascular abnormalities. Despite these changes, pHGG blood vessels retain some features of the BBB, as most endothelial cells express Glut1, and only a small subset co-express Plvap, a component of fenestrated pores [35]. This could be due to residual levels of Wnt signaling within gliomas, as BBB-specific Wnt-ligands (Wnt7a, Wnt7b and Norrin) are expressed by glial lineage cell-types, including oligodendrocyte progenitors and astrocytes [50–53]. It could also be due to variability in Vegf signaling, as Vegf ligands are required for the formation of fenestrated blood vessels in the choroid plexus [54]. While Wnt signaling is essential for sprouting angiogenesis into the developing CNS and BBB formation [36, 37, 49], how it interfaces with traditional pro-angiogenic factors like Vegf in development and pathological conditions remains an open area of investigation. Another possibility is that only certain mechanisms employed by the BBB are altered in pHGGs. While Glut1 expression is a BBB associated marker in CNS vasculature, its expression can be maintained in the presence of other NVU alterations that impact BBB function, such pericyte loss [55, 56]. Pericyte coverage has been shown to directly mediate transcytosis rates [55–57], and in healthy brain, receptor mediated transcytosis allows the selective crossing of plasma proteins, which switches to a more general transcytosis mechanism with aging and pericyte loss [58]. Decreased pericyte coverage in pHGGs could have a particular impact on transcytosis and could in part explain why Glut1 expression is maintained in regions that display vascular permeability.

Beside our immuno-staining characterization that demonstrates DMG blood vessels retain normal morphological features and BBB function, we find that DMG ECs maintain a transcriptional program similar to that of normal brain ECs. Examination of endothelial tip and stalk cell gene expression reveals increased expression of stalk cell genes in DMG ECs, and elevated expression of angiogenic tip cell genes in pHGG ECs. Moreover, PPI network analysis identified Sox17 as the most significantly enriched differentially expressed transcription factor in DMG ECs. Sox17 expression is highest in more mature brain endothelial cells [42], suggesting that tumor-associated blood vessels in DMGs mainly consist of existing mature vasculature, and not newly created vessels that develop in a highly organized fashion. Transcription factors enriched in pHGG EC PPI networks were mainly related to inflammatory mediated pathways, agreeing with the general immune-related signatures identified when comparing to normal or DMG ECs. Stat3 is a critical mediator of immune related responses in gliomas [59, 60]. Interactions between microglia and glioma tumor cells can promote a mesenchymal cell state in glioma tumor cells, which is dependent on Stat3 activation [61]. Additionally, increased cytokine expression caused by interactions between microglia and glioma cells can activate endothelial Jak / Stat3 signaling, resulting in increased vascular permeability in vitro [62]. Together, this can lead to increased endothelial expression of leukocyte adhesion molecules, which are associated with BBB dysfunction and inflammation [63, 64]. DMGs tend to display low T-cell infiltration compared to other gliomas [65]. Whether differences in the tumor microenvironment and pHGG and DMG vascular properties directly or indirectly influence the differential recruitment of infiltrating immune cells into tumors will be of particular interest to further delineate.

We find that DMG vessels are not particularly sensitive to the expression of secreted Wnt antagonists, which have previously been shown to drive BBB dysfunction in glioblastoma [16] and medulloblastoma [14, 15]. This finding, taken together with our data showing DMGs contain a stable network of blood vessels in a mature endothelial transcriptional state, lead us to postulate that differences in the angiogenic state of brain tumors plays a role in their responsiveness to fluctuations in Wnt signals. Canonical Wnt signaling by specific ligands (Wnt7a, Wnt7b, Ndp) and co-receptor complexes (Fzd4, Gpr124, Reck, Lrp5/6) is essential for BBB induction and maturation in the developing brain [36, 37, 46, 47, 66–69]. Yet, inhibition or deletion of these Wnt ligand or receptor components in the mature brain under normal homeostatic conditions does not impact vascular integrity or BBB function [17, 42]. Levels of endothelial Wnt signaling in the brain change over the course of brain development and maturation. Previous studies have shown canonical Wnt signaling, using the BAT (beta-catenin activated reporter) LacZ reporter mouse, decreases in brain endothelial cells as they mature [42, 70]. Moreover, expression of *Apcdd1*, an inhibitor of the canonical Wnt pathway, increases with age, ensuring the proper level of Wnt signaling required for proper angiogenesis and BBB development [71]. Understanding how these differences in Wnt signaling during vascular development and maturation apply to brain tumors will be important to consider not only for DMGs, but also for pHGGs and adult glioblastomas, since they also contain regions of tumor-associated vasculature that are not engaged in active angiogenesis.

In summary, we present a detailed analysis of pHGG and DMG tumor-associated vascular profiles, highlighting blood vessel heterogeneity and differences between these deadly brain tumors. Additionally, our data shows DMGs respond differently to variations in Wnt signaling levels, pointing out a need to further understand how canonical Wnt signaling and Vegf signaling interplay to regulate angiogenesis and BBB specification both in normal CNS development and in pathological settings. As current outcomes for most malignant gliomas are dismal, regardless of their vascular phenotype, there is a critical need for new and improved therapeutic strategies [8]. For example, strategies to “normalize” leaky and torturous blood vessels within brain tumors could be accomplished by stabilizing endothelial Wnt signaling. This could provide the benefit of promoting normal vascular growth and BBB function within tumors, enhancing the perfusion and vascularity of brain tumors even better than current anti-Vegf therapies. Recent strategies have leveraged receptor mediated transcytosis to deliver cargo into the brain [72, 73]. By defining the expression of receptors in endothelial cells across the normal brain and brain tumor types, one can develop approaches to target the delivery of new therapies into brain tumors that have been traditionally hard to penetrate. Transcytosis can also be upregulated using (microbubble-mediated) focused ultrasound (FUS). This is a non-invasive method that temporarily opens the BBB in a targeted location. Although drug delivery by FUS is thought to mainly function through mechanical stimulation of the blood vessels and consequent opening of tight-junctions, several other mechanisms of BBB opening, including transcytosis, have been described after ultrasound treatment [74]. Other methods to circumvent the BBB are convection-enhanced delivery (CED), in which drugs are directly infused into the parenchyma or tumor under a hydrostatic pressure gradient [75], the use of nanoparticles, or intranasal/intra-arterial delivery [76]. A better understanding of the tumor vasculature can help to decide which method to use in certain tumor types.

Together, the present work provides new insights that emphasize the need to consider vascular heterogeneity among brain tumors in the development of new therapeutic strategies.

## Supplementary Information

**Additional file 1** contains data related to supplementary figures 1–6. This includes vascular stains in (Fig S1) PDX and (Fig S2) primary pHGG and DMG samples, examples of intratumoral heterogeneity in IUE pHGG mouse models (Fig S3), validation of endothelial purity following magnetic cell sorting (Fig S4), PCA of endothelial samples (Fig S5), and validation of Dkk1 expression in IUE pHGG overexpression models (Fig S6).

**Additional file 2** contains data related to transcriptional analysis of endothelial samples from normal brain regions, IUE pHGG and IUE DMG mouse models. Tab1: DEGs comparing DMG vs. pHGG ECs; Tab2: DEGs comparing BS vs. DMG ECs; Tab3: DEGs comparing Ctx vs. pHGG ECs; Tab 4: Pathways enriched in pHGG ECs compared to DMG ECs; Tab5: Pathways enriched in DMG ECs compared to pHGG ECs.

## Data Availability

Supporting data for this manuscript are available in the Supplemental Information section. The RNA-sequencing data that support the findings of this study are available in GEO, deposited under the identifier GSE179372.

## Abbreviations

BBB: Blood brain barrier
DMG: Diffuse midline glioma
PHGG: Pediatric high-grade glioma
GEMMs: Genetically engineered mouse models
NVU: Neurovascular unit
CNS: Central nervous system
MRI: Magnetic resonance imaging
CE: Contrast enhancement
IUE: In utero electroporation
PDX: Patient derived xenograft
BS: Brainstem
CTX: Cortex
CB: Cerebellum
H&E: Hematoxylin and eosin
qPCR: Quantitative real-time polymerase chain reaction
TMR: Tetramethylrhodamine
ECs: Endothelial cells
Cldn5: Claudin5
Plvap: Plasmalemma vesicle associated protein
TF: Transcription factor
COLIV: Collagen IV
MACS: Magnetic cell sorting
FUS: Focused ultrasound
CED: Convection enhanced delivery
