# Supplementary material for: Defining tumor-associated vascular heterogeneity in pediatric high-grade and diffuse midline gliomas
Source: Acta Neuropathol Commun. 2021 Aug 23;9:142. doi: 10.1186/s40478-021-01243-1 (PMC8381557; doi:10.1186/s40478-021-01243-1)
Supplement: Supplementary file 1 — Additional file 1 contains data related to supplementary figures 1–6. This includes vascular stains in (Fig S1) PDX and (Fig S2) primary pHGG and DMG samples, examples of intratumoral heterogeneity in IUE pHGG mouse models (Fig S3), validation of endothelial purity following magnetic cell sorting (Fig S4), PCA of endothelial samples (Fig S5), and validation of Dkk1 expression in IUE pHGG overexpression models (Fig S6). [file 40478_2021_1243_MOESM1_ESM.pdf]

Additional File 1

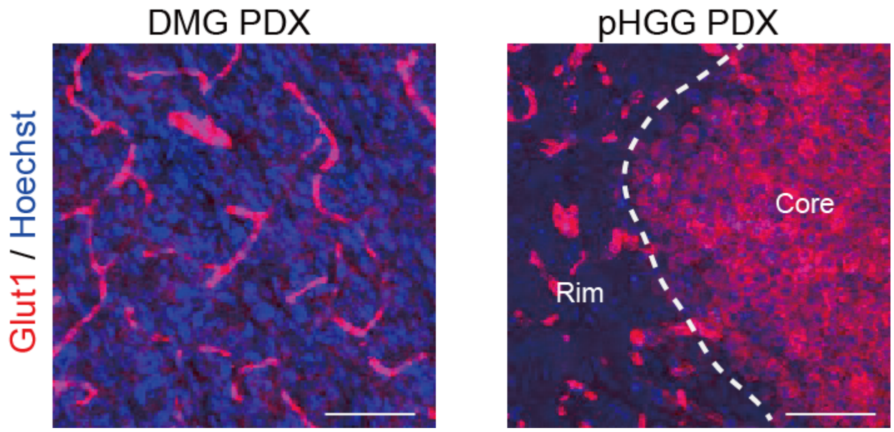

Fig. S1 – pHGG and DMG PDXs display tumor-associated vascular differences. Immunofluorescent images of Glut1 and Hoechst labeled pHGG and DMG PDX samples showing a core region of pHGG PDX with non-vascular Glut1 staining. Scale bars = 20µm.

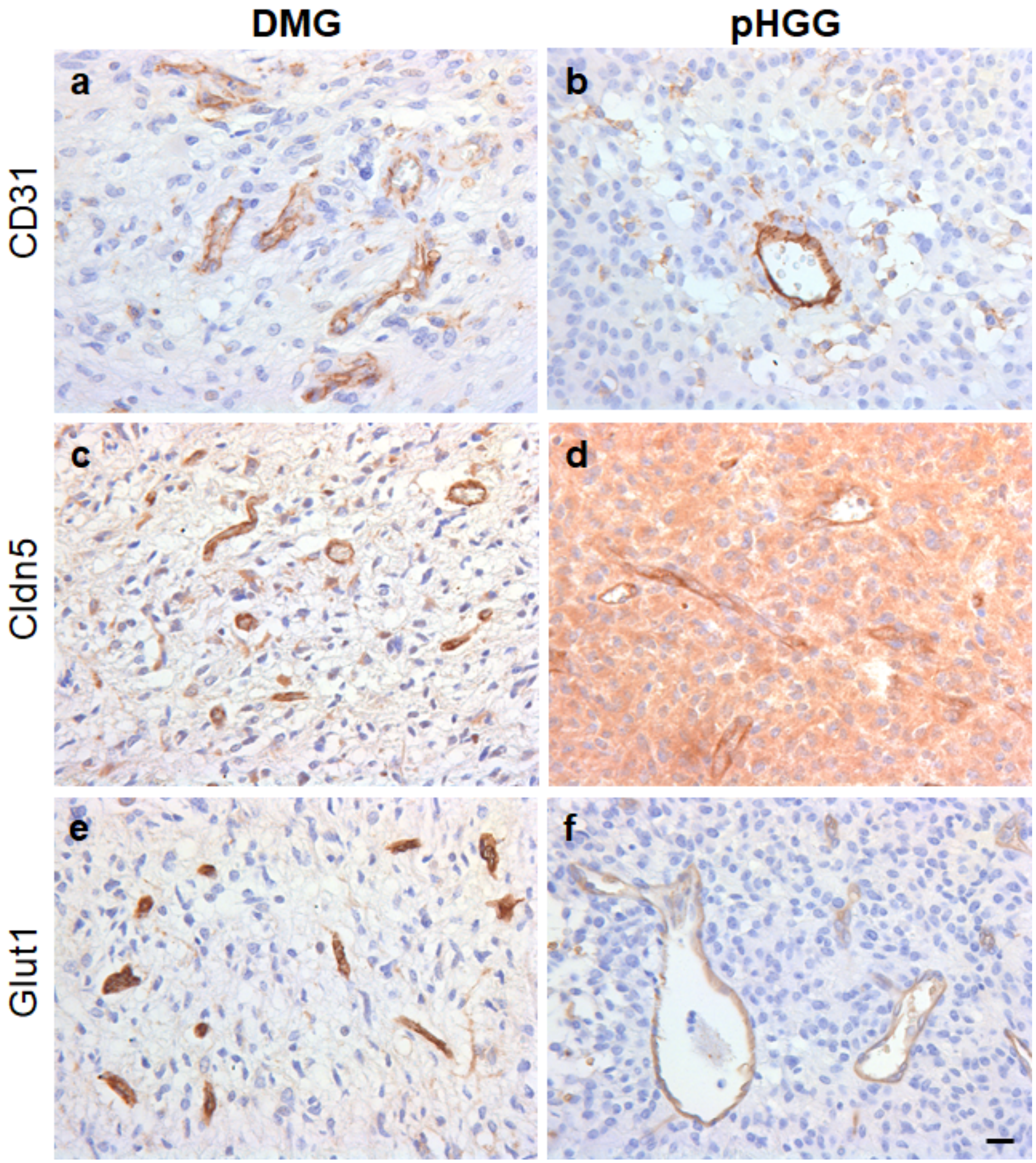

Fig. S2 – Primary DMG and pHGG human tumors recapitulate vascular differences of PDX models. (a, b) IHC staining for CD31 in DMG and pHGG samples. (c, d) IHC staining for Cldn5 in DMG and pHGG samples. (e, f) IHC staining for Glut1 in DMG and pHGG samples. Scale bar = 10μm

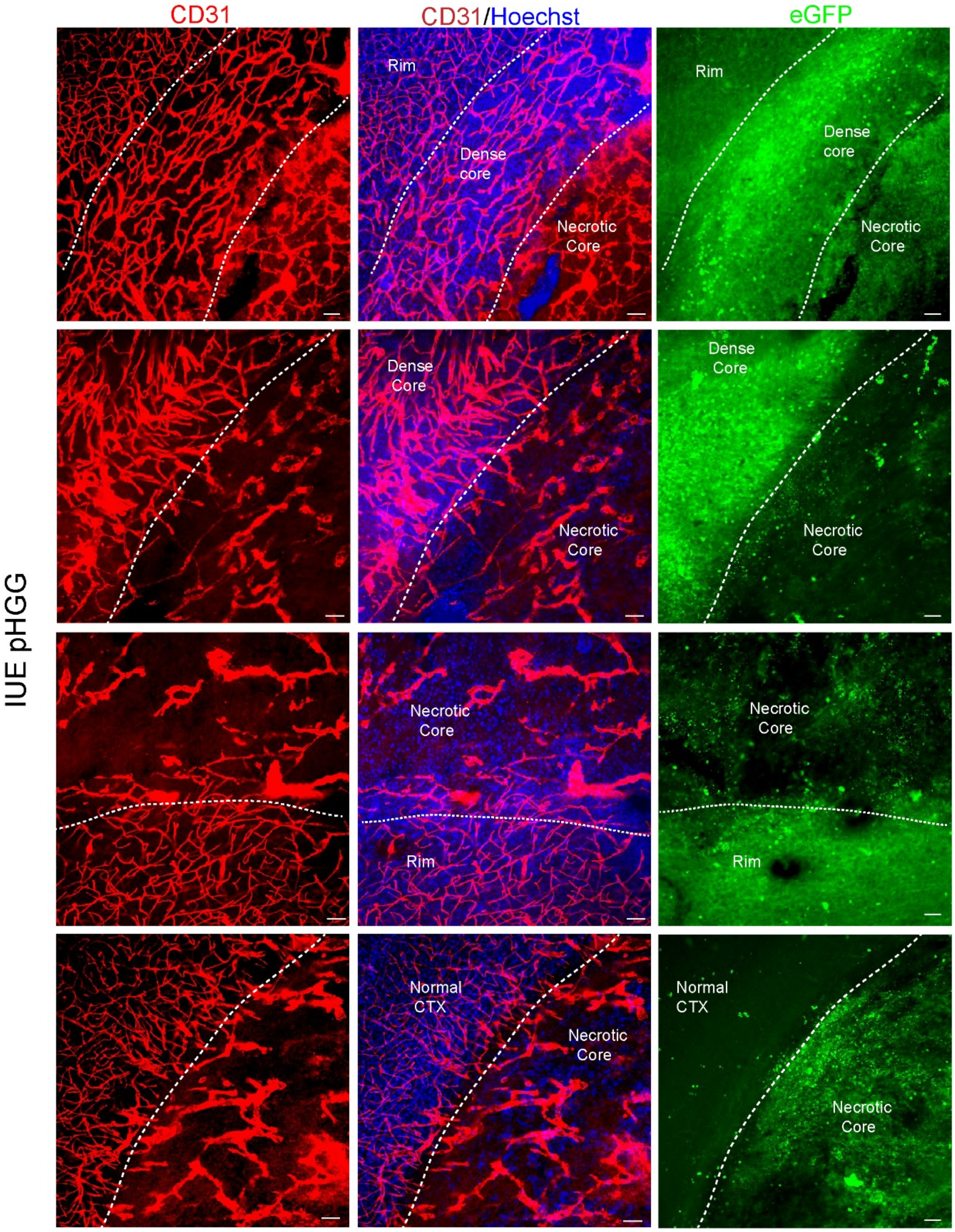

Fig. S3 – Intra-tumoral vascular heterogeneity within IUE pHGG mouse models. Representative immunofluorescent z-stack projection images from IUE pHGG tumors depicting

vascular heterogeneity of CD31-positive blood vessels across necrotic, dense tumor core, tumor rim, and normal brain regions. Scale bars = 20 μm.

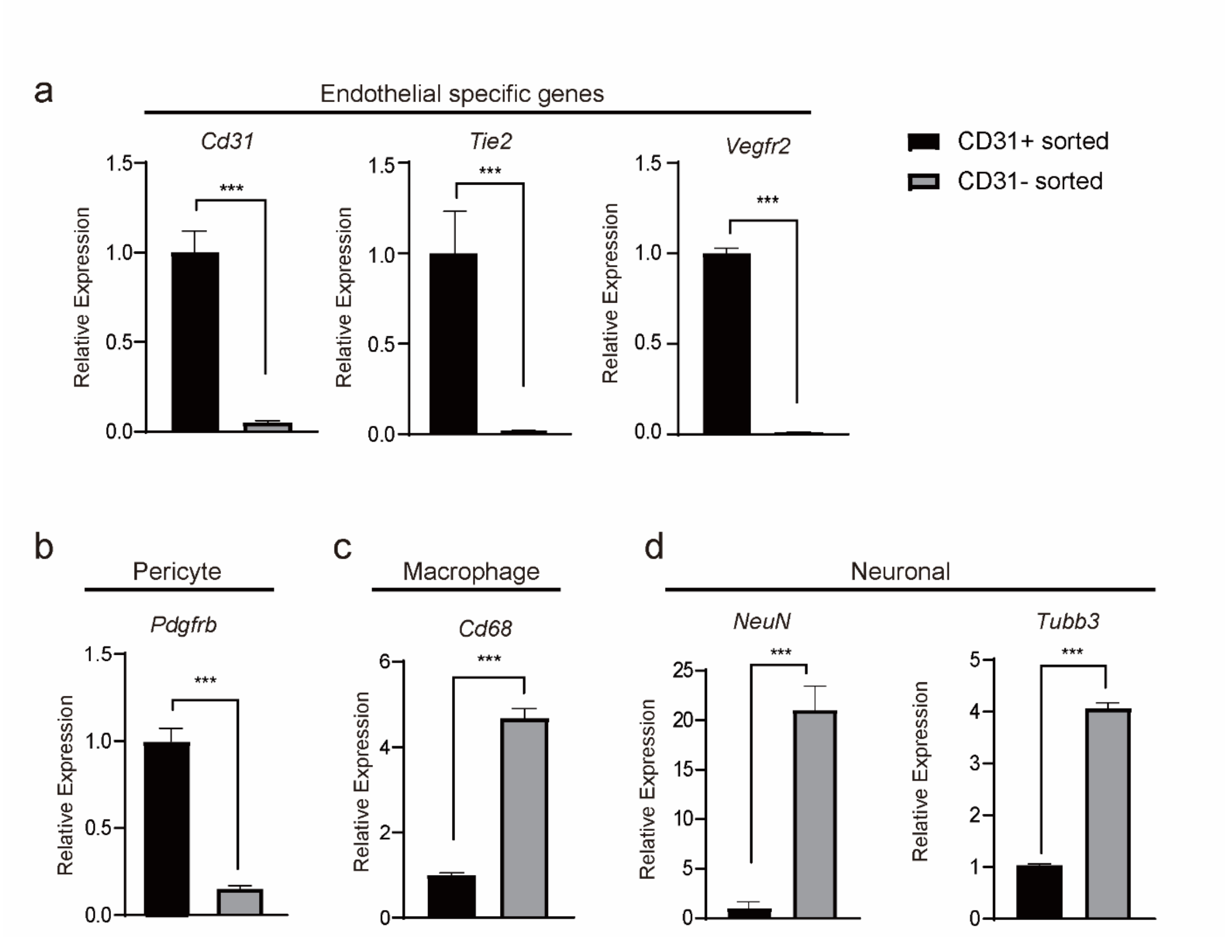

Fig. S4 – pHGG and DMG endothelial transcriptomes highlight heterogeneity of tumor-associated and normal brain endothelial signaling programs. (a-d) Validation of Cd31+/Cd45- endothelial fragments purified from control brainstem samples. qPCR analysis of endothelial (Cd31, Tie2, Vegfr2), pericyte (Pdgfrb), microglia/macrophage (Cd68) and neuron (NeuN, Tubb3) specific genes in CD31+CD45-ECs purified from normal brain tissues. (Data are presented as mean ± SEM. ***p<0.0001; unpaired t-test with Mann Whitney posthoc comparison).

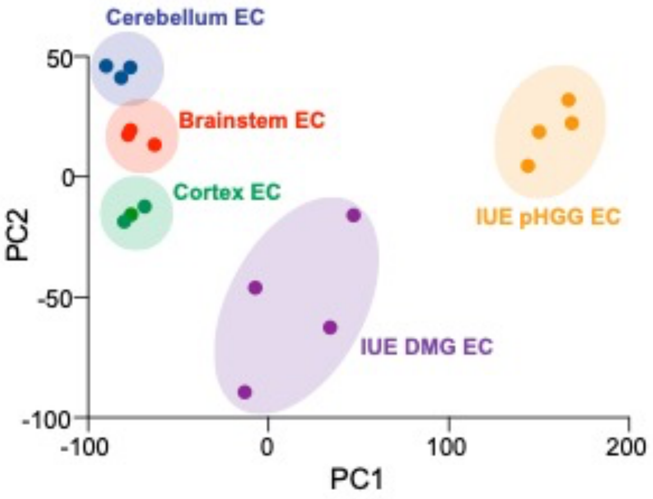

Fig. S5 – Principal Component analysis of endothelial samples. PCA of all endothelial samples based on transcriptome-wide expression.

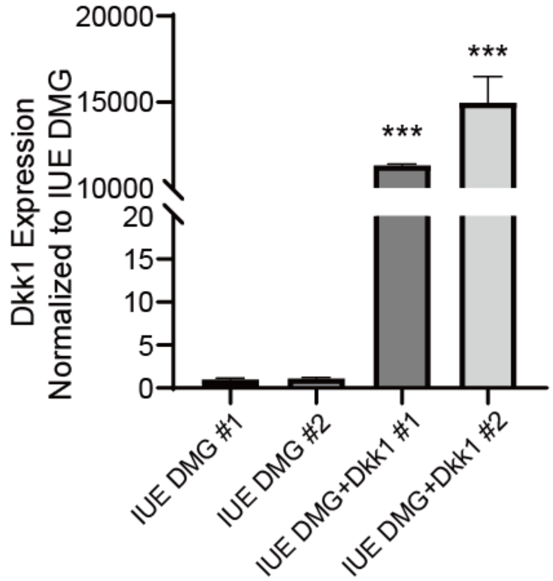

Fig. S6 – Confirmation of *Dkk1* expression in Dkk1 expressed DMG tumor tissues. qPCR of *Dkk1* in IUE DMG control and IUE DMG+Dkk1 samples (Data are presented as mean ± SEM. ***$p<0.0001$; unpaired t-test).
